# The Effect of miR-361-3p Targeting TRAF6 on Apoptosis of Multiple Myeloma Cells

**DOI:** 10.4014/jmb.2010.10059

**Published:** 2020-12-14

**Authors:** Zhen Fan, Zhiwei Wu, Bo Yang

**Affiliations:** Department of Hematology, The First People’s Hospital of Jingmen, No. 67 Xiangshan Avenue, Jingmen, Hubei Province 448000, P.R. China

**Keywords:** miR-361-3p, multiple myeloma, tumor necrosis factor receptor-associated factor 6, apoptosis

## Abstract

microRNA-361-3p (miR-361-3p) is involved in the carcinogenesis of oral cancer and pancreatic catheter adenocarcinoma, and has anti-carcinogenic effects on non-small cell lung cancer (NSCLC). However, its effect on multiple myeloma (MM) is less reported. Here, we found that upregulating the expression of miR-361-3p inhibited MM cell viability and promoted MM apoptosis. We measured expressions of tumor necrosis factor receptor-associated factor 6 (TRAF6) and miR-361-3p in MM cells and detected the viability, colony formation rate, and apoptosis of MM cells. In addition, we measured expressions of apoptosis-related genes Bcl-2, Bax, and Cleaved caspase-3 (C caspase-3). The binding site between miR-361-3p and TRAF6 was predicted by TargetScan. Our results showed that miR-361-3p was low expressed in the plasma of MM patients and cell lines, while its overexpression inhibited viability and colony formation of MM cells and increased the cell apoptosis. Furthermore, TRAF6, which was predicted to be a target gene of miR-361-3p, was highexpressed in the plasma of patients and cell lines with MM. Rescue experiments demonstrated that the effect of TRAF6 on MM cells was opposite to that of miR-361-3p. Upregulation of miR-361-3p induced apoptosis and inhibited the proliferation of MM cells through targeting TRAF6, suggesting that miR-361-3p might be a potential target for MM therapy.

## Introduction

Multiple myeloma (MM) is a plasma cell malignancy caused by aggregation of monoclonal plasma cells and has a hereditary complexity [17, 20, 25]. Statistics showed that [22] MM accounts for 10% of all hematological malignancies and 1% of all cancer cases with an annual incidence of 4.5-6.0/100,000 in Europe. The average age of patients is 72 at the time of their diagnosis and the annual mortality rate is 4.1/100,000. Though treatment of MM has greatly improved, difficulties still remain. Therefore, it is highly necessary to further explore the molecular mechanism of the development and progression of MM.

MicroRNAs (miRNA) are single-stranded non-coding RNAs (18-25 nucleotides in length) that play significant roles in development, aging, and diseases as well as in the formation of transcripts of many cell types [33]. miRNAs have been increasingly found to play regulatory roles in some cancer cells. Ling H. *et al*. found that miR-224 promotes colorectal cancer metastasis [18]. Yonemori M. *et al*. [31] showed that miR-139-5p and miR-139-3p are downregulated in clinical specimens of bladder cancer and the two anti-cancer effects in bladder cancer cells. miR-24-3p was found to be upregulated in breast cancer tissues and predicted a lower survival rate of patients with the cancer [15]. miRNAs also regulate cancer promotion and have anti-cancer effects, which involve a variety of mechanisms. For example, cell cycle arrest of colon cancer cells at G1 phase is induced by small RNA of the miR-6883 family targeting CDK4/6 [19]. miR-146 could promote the survival of cervical cancer cells by targeting tumor necrosis factor receptor-associated factor 6 (TRAF6) [12]. TRAF6 is involved in many biological processes including innate immune system operation and bone formation [29]. According to reports, TRAF6 is a TLR effector with ubiquitin ligase activity and is overexpressed in hematopoietic stem/progenitor cells of myelodysplastic syndromes. Moreover, overexpression of TRAF6 in mouse hematopoietic stem/progenitor cells impairs hematopoietic function and causes bone marrow failure [8]. In addition to these relationships between diseases and miRNAs, evidence has increasingly indicated that some abnormally expressed miRNAs are related to survival and proliferation of MM cells [3, 21].

miR-361-3p has a variety of biological functions and a critical role in many diseases. Hu J. *et al*. [11] demonstrated that miR-361-3p was a small tumor molecule in promoting metastasis and the miR-361-3/DUSP2/ERK axis is a novel epithelial-to-mesenchymal transition (EMT) axis in pancreatic ductal adenocarcinoma. Similarly, Chen W. *et al*. [7] found that miR-361-3p could inhibit tumor growth in NSCLC and its anti-tumor activity might be related to the inhibition of target gene SB2B1. From the above studies, it could be speculated that abnormal expression of miR-361-3p is involved in a variety of cancers. Previous study [4] also indicated that a total of 1,799 miRNAs were found abnormally expressed in MM samples and among them, miR-361-3p was down regulated. However, the effect and potential mechanisms of miR-361-3p in MM are less reported. Thus, in this study, we explored the biological behaviors of miR-361-3p in MM cells and the potential mechanism.

## Materials and Methods

### Patient Samples

From May 2018 to February 2019, the plasma of bone marrow blood was obtained from 30 MM patients (newly diagnosed or relapsed) ranging in age between 29 to 72 years old and from 30 healthy volunteers aged between 27 and 74 years old. Patients meeting the following CRAB criteria [16] of MM were included: renal insufficiency, hypercalcemia (>11.5 mg/dL), and anemia (hemoglobin<10 g/dL or 2 g/dL< normal) with bone lesions. Healthy human normal plasma (nP) was obtained as a normal control in the following study. The study was approved by the Ethics Committee of the First People’s Hospital of Jingmen (No. 201805283615). Written informed consent was obtained from each patient to the use of their samples in the scientific research when sample collection took place.

### Cell Lines and Cell Culture

MM cells (MM1S, RPMI-8226, H929, U266) were purchased from the American Type Culture Collection (ATCC, USA). The cells were cultured in a Roswell Park Memorial Institute 1640 (RPMI-1640) medium (Gibco Laboratories, USA) supplemented with 1% penicillin/streptomycin (Sigma-Aldrich, USA) and 10% fetal bovine serum (FBS) (Gibco, Australia) in an incubator at 37°C with 5% CO_2_. When the cells reached approximately 75%confluence, they were subcultured and those in the logarithmic phase were taken for experiment.

### Cell Transfection and Grouping

The miR-361-3p mimic (mimic) and mimic control were obtained from RIBOBIO (China). The mimic sequences were 5′-CCCCCAGGUGUGAUUCUGAUUUGC-3′ (sense), 5′-AAAUCAGAAUCACACCUG GGGGUU-3′ (antisense). Sequences of mimic control were (5′-UUCUCCGAACGUGUCACGUTT-3′ (sense) and 5′-ACGUGACACGUUCGGAGAATT-3′ (antisense). TRAF 6 gene was inserted into pcDNA3.1 vector (Genepharma) to construct TRAF 6 overexpression vector pcDNA3.1-TRAF6. pcDNA3.1 vector was used as negative control (NC). MM1S and U266 cells were cultured into 60% confluence and then transfected with oligonucleotides using Lipofectamine 2000 (Invitrogen, USA). Next, transfection efficacy was evaluated by performing quantitative real-time reverse transcription PCR (qRT-PCR) analysis. Further experiments were carried out at 48 h after the transfection. Each experiment was performed in triplicate.

To further analyze the effects of miR-361-3p on the cell biological behaviors (i.e., cell proliferating, colony, and apoptosis of MM cells), the cells were divided into blank (untreated cells), mimic control, and mimic. For exploring the effects of TRAF6 on the proliferation, colony formation, and apoptosis of MM cells, the cells were divided into mimic control+NC, mimic control+TRAF6, mimic+NC, and mimic+TRAF6.

### qRT-PCR Analysis

Total RNAs were extracted from MM1S and U266 cells using TRIzol reagent (Invitrogen). For detecting miRNA expression, cDNAs were synthesized using TaqMan miRNA Assays (Applied Biosystems, China) and expressions of mRNAs were determined using M-MLV reverse transcriptase (BioTeke, China). The qRT-PCR reaction was performed in an Applied Biosystems AB 7500 Real-Time PCR system (Invitrogen) using SYBR Green PCR Master Mix (Toyobo, Japan) following the manufacturers’ instructions. The primers were as follows: miR-361-3p forward, 5'-UCCCCCAGGUGUGAUUCUGAUUU-3' and miR-361-3p reverse, 5'-GCAAAT CAGAATCACACCTG-3'; GAPDH forward, 5'-CCATGTTCGTCATGGGTGTG-3' and GAPDH reverse, 5'-GGTG CTAAGCAGTTGGTGGTG-3'; U6 forward, 5'-CGCTTCGGC AGCACATATACTA A-3' and U6 reverse, 5'-TATGGA ACGC TTCACGA ATTTGC-3'; TRAF6 forward, 5'- AGACAAGACCATCAAATCCGGGAG -3' and TRAF6 reverse, 5'-TCCAGGGCTATGAATCACAACAGG-3'. GAPDH and U6 served as internal reference of mRNA and miR-361-3p, respectively. The relative gene expressions were calculated and quantified using the 2-ΔΔCt method [28]. Conditions for RT reaction were as follows: at 37°C or 60 min, and at 85°C for 5 min. After the RT reaction, 2 μl cDNA products diluted to 1:5 were used to perform qRT-PCR reactions at 95°C for 10 min, 40 cycles at 95°C for 10 sec, at 60°C for 30 sec, and at 72°C for 30 sec.

### Cell Counting Kit-8 (CCK-8) Assay

CCK-8 assay was performed to detect the viability of cells. First, 100 μl MM1S and U266 cell suspension were cultured in 96-well plates (1 × 10^3^ cells/well) and added with CCK-8 reagent (10 μl/well) at 24, 48, and 72 h. The cells were further incubated at 37°C for 1 h. Then, the absorbance at 450 nm was detected using a microtiter plate (Thermo Labsystems, USA).

### Colony Formation Assay

MM1S and U266 cells were seeded into 6-well plates (200 cells/well) and maintained in culture media containing 0.3% noble agar at 37°C for 14 days. The culture medium was then removed and the cells were washed twice using PBS buffer. Then, the cells were immobilized by 100% methanol for 30 min, the methanol was removed, and the cells were washed twice by PBS buffer again, and then stained by 0.1% crystal violet (Sigma-Aldrich). The colonies number was observed under a microscope (Olympus Corp., Japan) to calculate the colony formation rates.

### Apoptosis Assay

To study cell apoptosis, propidium iodide (BioVision, USA) double staining and Annexin V-fluorescein isothiocyanate staining were performed according to the manufacturer’s instructions. The cell apoptosis was detected by flow cytometric analysis (BD FACSCanto II Flow, Beckman-Coulter, USA). The cells in the upper left area are cells after necrosis, which was reduced to 0% by optimizing operations, such as the speed and time of centrifugation, selection of apoptosis kit, or the door of FSC/SSC.

### Western Blot (WB) Analysis

MM1S and U266 cells were lysed by ice-cold lysis buffer. Equal amounts of proteins were separated and transferred onto a polyvinylidene fluoride (PVDF) membrane at 100 V for 1.5 h. After blocking the membrane with 5% non-fat milk for 1 h, the blots were incubated with primary antibodies specific to Bcl-2 (1:1000, 26 kD, ab59348, Abcam), Bax (1:1000, 21 kD, ab32503, Abcam), Cleaved-caspase-3 (C caspase-3) (1:1000, 17 kD, ab2302, Abcam), and TRAF6 (58 kD, 1:1000, ab33915, Abcam) at 4°C overnight. Then, the membrane was further incubated with horseradish peroxidase-conjugated secondary antibody (1:2000; Abcam) at room temperature for 2 h. The expression levels of proteins were detected using Image-Pro Plus 6.0 software (Media Cybernetics, Inc., USA). GAPDH served as an internal control.

### Dual-Luciferase Reporter Assay

The TRAF6 fragments containing the presumed or mutant binding site of miR-361-3p were amplified by PCR and cloned into pmirGLO double luciferase miRNA target expression vector (Promega, USA) to obtain TRAF6-Wild-Type (TRAF6-WT) and TRAF6-Mutated-Type (TRAF6-MUT). MM1S and U266 cells were co-transfected with TRAF6-WT or TRAF6-MUT and blank or miR-361-3p mimic using Lipofectamine 2000 (Life Technologies, USA). The luciferase activity was detected by performing dual-luciferase reporter assay (Promega) 48 h after the cell transfection.

### Statistical Analysis

SPSS 20.0 software (SPSS Inc., USA) was used to analyze the data, which were expressed as mean ± standard deviation. Student’s *t*-test was used to analyze the difference between the two groups. One-way ANOVA followed by Tukey’s test was used to analyze the difference of two or more experimental groups. *p*-values < 0.05 was considered as statistically significant.

## Results

### The miR-361-3p Expression Level in Plasma from Bone Marrow Blood of MM Patients and MM Cell Lines and Its Effect on Cell Viability

**Fig. 1:**
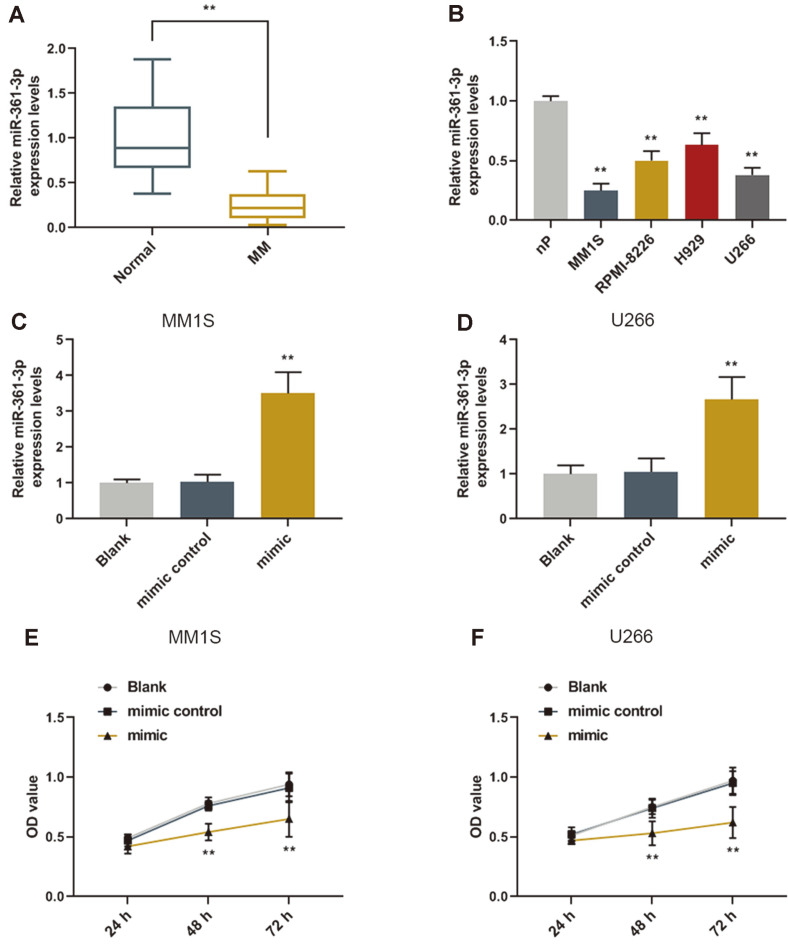
The miR-361-3p expression in plasma and cell lines of MM patients and its effect on cell viability. (**A**) The qRT-PCR results revealed that miR-361-3p in plasma of patients with MM was remarkably down regulated. **p < 0.001 vs. normal. (**B**) miR-361-3p in MM cell lines was down regulated. **p < 0.001 vs. nP. (**C**-**D**) The expression level of miR-361-3p was up regulated in the cells transfected with miR-361-3p mimic. (**E**-**F**) The CCK-8 assay results revealed that cells transfected with miR-361-3p mimic had a lower viability. **p < 0.001 vs. mimic control.

The miR-361-3p in plasma from bone marrow blood of patients with MM was remarkably down regulated compared with that of normal controls (Fig. 1A). Moreover, the expressions of miR-361-3p were decreased in MM cell lines (MM1S, RPMI-8226, H929, U266) compared with normal controls (nP) (Fig. 1B). The transfection assay results revealed that cells transfected with miR-361-3p mimic had a higher expression level of miR-361-3p compared with that of mimic control, with a limitation not showing positive control for PI negative (Figs. 1C-1D). Furthermore, the CCK-8 assay results showed that miR-361-3p overexpression inhibited viability of MM cells (Figs. 1E-1F).

### miR-361-3p Affected Colony and Apoptosis of MM Cells

**Fig. 2:**
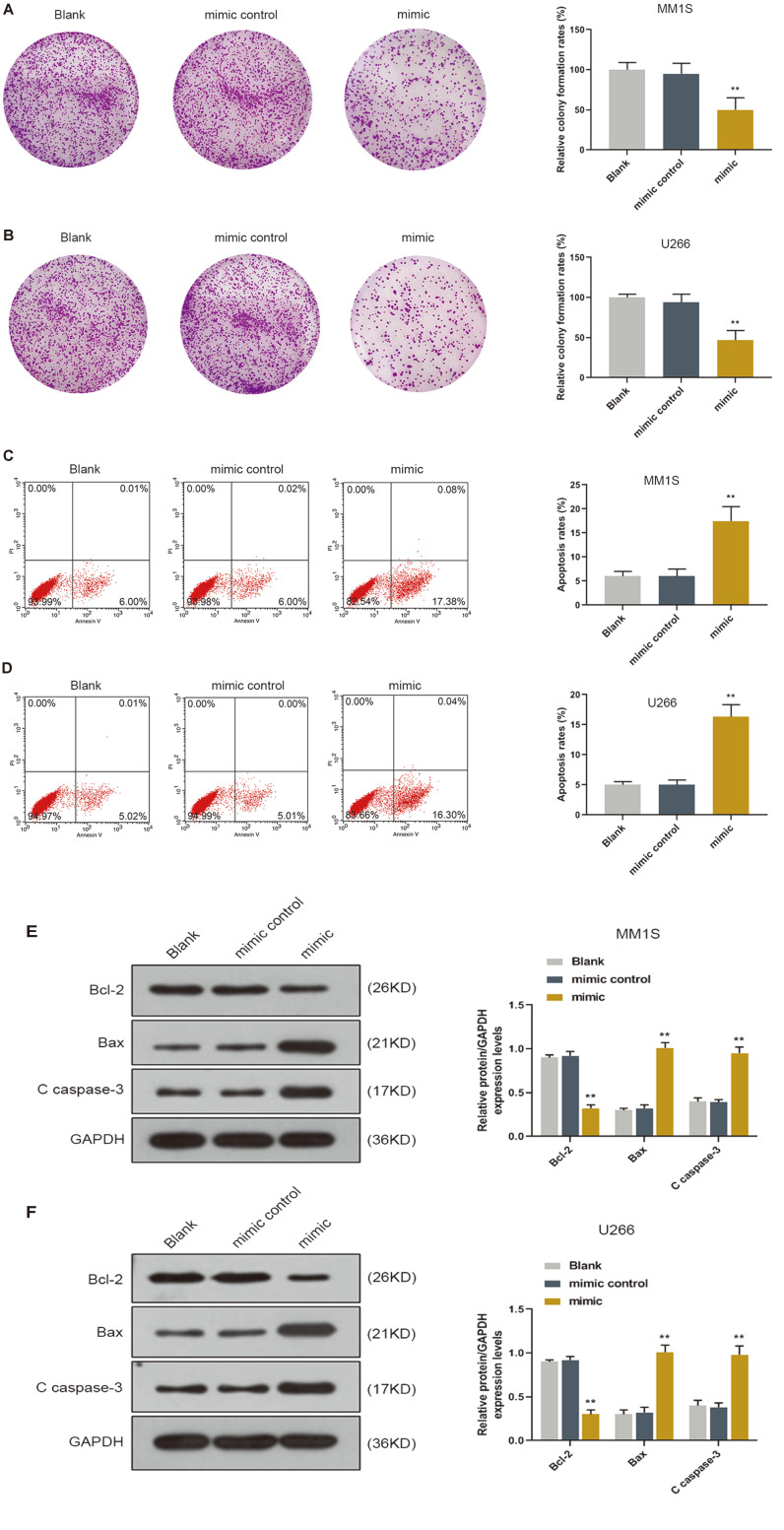
Effects of miR-361-3p on colony and apoptosis of MM cells. (**A**-**B**) The colony formation assay results showed that miR-361-3p overexpression inhibited the colony formation rate of cells. (**C**-**D**) The apoptosis assay results revealed that overexpression of miR-361-3p promoted the apoptosis of cells. (**E**-**F**) The western blot (WB) showed that expressions of Bax and C caspase-3 were up regulated and Bcl-2 expression was down regulated in cells. **p < 0.001 vs. mimic control.

After the successful transfection of miR-361-3p mimic and mimics control, the colony formation assay results showed that miR-361-3p overexpression inhibited the colony formation of cells compared with that of the mimic control group (Figs. 2A-2B). In addition, overexpression of miR-361-3p induced apoptosis of the MM cells (Figs. 2C-2D). Moreover, the WB analysis results demonstrated that the expressions of Bax and C caspase-3 were higher and Bcl-2 expression was lower in the cells transfected with miR-361-3p mimic, as compared with those in mimic control group (Figs. 2E-2F).

### TRAF6 Was a Target Gene of miR-361-3p and Its Effects on Viability of MM Cells

**Fig. 3:**
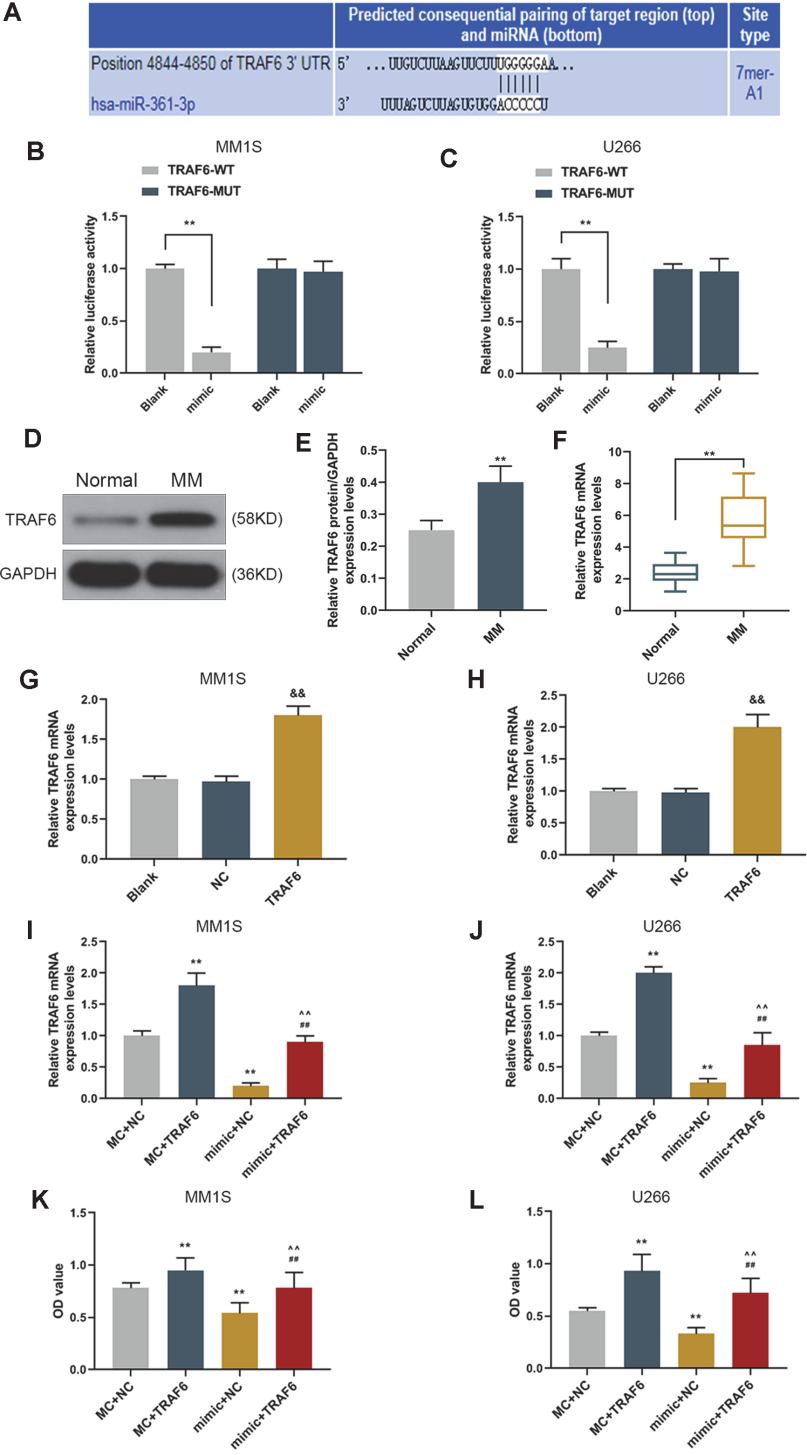
TRAF6 was a target gene of miR-361-3p and the effects of TRAF6 on cell viability. (**A**) TRAF6 was the possible target genes of miR-361-3p, predicted by TargetScan. (**B**-**C**) Dual-luciferase reporter assay results revealed that the miR-361-3p mimic reduced luciferase activity of TRAF6 -WT. **p < 0.001 vs. Blank. (**D**-**F**) The WB and qRT-PCR analysis result showed that TRAF6 was more up regulated in plasma of MM patients than that of normal controls. **p < 0.001 vs. Normal. ##*p* < 0.001 vs. normal 2. (**G**-**H**) The qRT-PCR analysis was applied to show the increased TRAF6 expression levels after TRAF6 overexpression transfection. (**I**-**J**) The qRT-PCR analysis result showed that overexpression of miR-361-3p down regulated the level of TRAF6. (**K**-**L**) The CCK-8 assay results revealed that TRAF6 overexpression increased cell viability, which partly reversed the inhibitory effect of miR-361-3p overexpression on MM cells. &&*p* < 0.001 vs. NC; **p < 0.001 vs. MC+NC; ##*p* < 0.001 vs. MC+TRAF6; ^^*p* < 0.001 vs. mimic+NC. MC was mimic control, NC was the empty control for TRAF6.

TRAF6 is involved in many biological processes including innate immune system operation and bone formation. The mechanism of the effects of miR-361-3p on the MM cells was investigated. TargetScan (http://www.targetscan.org/vert_71/) predicted that there were binding sites between miR-361-3p and TRAF6 (Fig. 3A). The prediction was further verified by performing dual-luciferase reporter assay. We found that miR-361-3p mimic reduced luciferase activity of TRAF6 -WT but did not affect that of TRAF6-MUT (Figs. 3B-3C). TRAF6 was therefore confirmed as a target gene of miR-361-3p exerting its effects on the viability of MM cells. The mRNA and TRAF6 levels from 30 MM patients and 30 normal people were analyzed by WB and qRT-PCR and the results showed that TRAF6 was more up regulated in the plasma of MM patients than that of normal controls. The representative two pairs of western blots (each group were of samples from 10 different people in normal or MM group) were exhibited (Figs. 3D-3F). The qRT-PCR analysis was applied to show the increased TRAF6 expression levels after TRAF6 overexpression transfection (Figs. 3G-3H). Furthermore, the results also showed that the expression level of TRAF6 of the cells transfected with miR-361-3p mimic was reduced (Figs. 3I-3J). Moreover, the CCK-8 assay data indicated that TRAF6 overexpression partly reversed the inhibitory effect of cell viability of MM cells by miR-361-3p overexpression (Figs. 3K-3L).

### The Effects of TRAF6 on Colony and Apoptosis of MM Cells

**Fig. 4:**
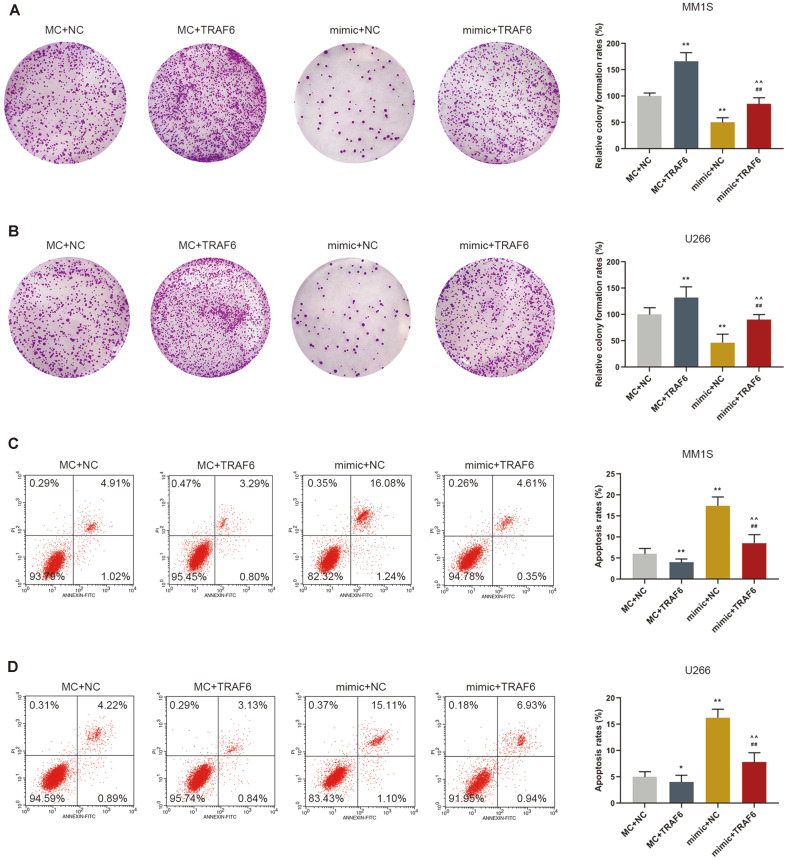
The effects of TRAF6 on colony and apoptosis of MM cells. (**A**-**B**) The colony formation assay results revealed that overexpression of TRAF6 promoted the colony formation of cells. (**C**-**D**) The apoptosis assay results showed that overexpression of TRAF6 reduced the apoptosis of cells. **p < 0.001 vs. MC+NC; ^##^*p* < 0.001 vs. MC+TRAF6; ^^*p* < 0.001 vs. mimic+NC.

We further explored the effects of TRAF6 on colony and apoptosis of MM cells. The colony formation assay results revealed that overexpression of TRAF6 promoted the colony formation of the MM cells, as compared with those of the cells transfected with miR-361-3p mimic (Figs. 4A-4B). Moreover, the apoptosis assay results showed that overexpression of TRAF6 reduced the apoptosis rate of the cells and partly reversed the cell apoptosis rate of the MM cells increased by miR-361-3p mimic transfection, with a limitation not showing positive control for PI negative (Figs. 4C-4D).

## Discussion

MM is an advanced clonal B-cell disease and recently, studies on the molecular pathogenesis and biology of MM demonstrated the complexity of epigenomics in the pathogenesis, prognosis and high individual variability of MM [2]. Genetic abnormalities, which are involved in pathogenesis and therapeutic resistance of MM, mainly affect DNA methylation/histone modification patterns of genes and miRNAs [24]. Thus, understanding the molecular mechanism related to MM is of great significance for exploring the treatment of this disease.

miRNAs play important roles in cancers and previous study [27] showed that LNA/DNA gapmers specific to miR-17-92 pri-miRNA has an anti-tumorous activity by inhibiting the maturation of the miR-17-92 pri-miRNA, subsequently downregulating all the cluster members. Down regulation of miRNA-29b could inhibit the growth and survival of MM cells. Researchers found that miR-29b overexpression affects pro-tumor and pro-inflammatory potentials of dendritic cells through various molecular mechanisms and reduces the cells’ ability to support the growth of MM cells [5]. Similarly, miR-1271 could promote apoptosis and inhibit MM cell proliferation via inhibiting the Smoothened-mediated Hedgehog signaling pathway [30]. According to research, long non-coding RNA (LncRNA) Malat1 regulates Foxp1 expression by activating mir-509-5p, thus acting as an oncogene in MM [10]. MiR-144-3p was also found to induce apoptosis and inhibit proliferation of MM cells via targeting c-Met [34].

MiR-361-3p has certain biological effects on different cancers and a recent study [32] found that miR-361-3p plays an anti-cancerous role in the occurrence and development of retinoblastoma by targeting sonic Hedgehog signaling. However, little is known about the potential molecular mechanisms of miR-361-3p in MM. Here, we investigated the effects of miR-361-3p on proliferation, cloning, and apoptosis of MM cells through a series of in vitro cell experiments and also analyzed the possible mechanism of these effects. The results showed that miR-361-3p regulated the biological functions of MM cells such as apoptosis and was related to targeting TRAF6. The limitation was not detecting the expression of miR-361-3p and TRAF6 mRNA in plasma cells of MM patients.

We found that miR-361-3p expression level of plasma of patients with MM was remarkably down regulated and similar findings were also observed in the MM cell lines. From these results, we speculated that abnormal expression of miR-361-3p in MM cells may be related to the biological behaviors of the cells. The results of CCK-8 assay, colony formation assay, transwell assay, wound-healing assay and apoptosis assay confirmed our speculation as we found that miR-361-3p overexpression inhibited the proliferation of MM cells, and promoted the apoptosis of MM cells.

According to Tang L. *et al*. [26], miR-361-3p is low-expressed in murine spermatogonia and in the murine GC-1 spermatogonia cell lines in vivo and ultimately caused apoptosis of spermatogonia. In order to investigate the effects of miR-361-3p on MM cells, we also studied the effects of miR-361-3p overexpression on Bcl-2, Bax, and C-caspase-3. Under various stimuli, cell apoptosis may occur, and this process begins with the activation of “initiator” caspases, which divides and activates the “executioner” caspases [6]. Research revealed that Bcl-2 is an inhibitor of apoptosis and cytokines transmit signals of cell survival and proliferation through different ways [13], and that abnormal apoptosis can lead to malignant transformation [1]. Karpel-Massler G. *et al*. [14] indicated that inhibition of Bcl-2 helps reduce synthetic lethality of tumors to therapy. In contrast to Bcl-2, Bax is a key protein in the pro-apoptotic family and can promote the permeability and release of ROS and activates apoptotic cascade reaction [29]. The current findings showed that miR-361-3p overexpression induced apoptosis of MM cells. This discovery encouraged us to further explore the mechanism of miR-361-3p on MM cells.

TargetScan predicted that there were binding sites between miR-361-3p and TRAF6 and the prediction was further verified by performing dual-luciferase reporter assay. Furthermore, compared with the normal controls, MM patients had a higher TRAF6 expression level. Fang J. *et al*. [9] showed that the absence of Traf6 reduces the self-renewal ability of hematopoietic stem cells, and results in premature death and hematological defects. A recent study [23] reported that activating the NFκB pathway is the key to the pathogenesis of MM and TRAF6 has been previously found to act as an important mediator in NFκB activation. Through rescue experiments, we discovered that the effect of TRAF6 on cells was opposite to that of miR-361-3p on the MM cells. To conclude, we found that miR-361-3p affects the apoptosis of MM cells mainly through targeting TRAF6 gene.

The relationship between the type of myeloma and the level of mir-361-3p or TRAF6 was not studied in this article, which might be a limitation and should be studied more in the future.

Our findings revealed that overexpression of miR-361-3p affects the biological behaviors of MM cells. Specifically, overexpressed miR-361-3p inhibits colony formation of MM cells and promotes the cell apoptosis. TRAF6 is predicted to be a target gene to miR-361-3p and the abovementioned effects of miR-361-3p on MM were realized through targeting TRAF6. However, the current study also has some limitations. For example, whether the regulation of miR-361-3p targeting TRAF6 on the biological functions of multiple myeloma is related to other genes or pathways still needs further studying in vitro and in vivo.
